# Atopic Dermatitis and Autism Spectrum Disorders: Common Role of Environmental and Clinical Co-Factors in the Onset and Severity of Their Clinical Course

**DOI:** 10.3390/ijms25168936

**Published:** 2024-08-16

**Authors:** Rossella Casella, Andrea Miniello, Federica Buta, Mona-Rita Yacoub, Eustachio Nettis, Giovanni Pioggia, Sebastiano Gangemi

**Affiliations:** 1 Department of Emergency and Organ Transplantation, School of Allergology and Clinical Immunology, University of Bari Aldo Moro, Policlinico di Bari, 70120 Bari, Italy; 2 School and Division of Allergy and Clinical Immunology, Department of Clinical and Experimental Medicine, University of Messina, 98122 Messina, Italy; federicabuta@gmail.com (F.B.);; 3 Unit of Immunology, Rheumatology, Allergy and Rare Diseases, IRCCS Hospital San Raffaele, 20132 Milan, Italy; 4 Institute for Biomedical Research and Innovation (IRIB), National Research Council of Italy (CNR), 98164 Messina, Italy

**Keywords:** atopic dermatitis, autism spectrum disorders, ADHD, genetic, exposome

## Abstract

Increasing evidence suggests an association between atopic dermatitis, the most chronic inflammatory disease of the skin, and autism spectrum disorders, which are a group of neurodevelopmental diseases. Inflammation and immune dysregulation associated with genetic and environmental factors seem to characterize the pathophysiological mechanisms of both conditions. We conducted a literature review of the PubMed database aimed at identifying the clinical features and alleged risk factors that could be used in clinical practice to predict the onset of ASD and/or AD or worsen their prognosis in the context of comorbidities.

## 1. Introduction

Atopic dermatitis (AD) is the most common chronic inflammatory disease of the skin, and affects up to 20% of children worldwide [1]. It is characterized by intense itching and excoriations with erythematous, xerotic, fissured skin and lichenification [2]. The first clinical manifestations may be observed during childhood, and the early onset of AD typically characterizes more severe disease [1]. AD is generally associated with other atopic conditions, such as asthma, allergic rhinitis and chronic rhinosinusitis with nasal polyps [3]. The pathogenesis of AD has not been fully clarified; however, major risk factors for AD are mutations in filaggrin genes, which are associated with a defect of the epithelial barrier that contributes to the disease flaring in response to environmental stimuli [4].

Increasing evidence from the literature suggests an association between AD, autism spectrum disorders (ASDs) and attention deficit hyperactivity disorder (ADHD). ASD, commonly named autism, refers to a group of neurodevelopmental diseases, characterized by impairments in interaction and communication skills and stereotypical sensory–motor schemes [5].

In the last few decades, ASD has gained increasing attention from the scientific community, which has contributed to early diagnoses and thus interventions [6]. However, a diagnosis of ASD can still be very challenging, due to its heterogeneous clinical presentation and the lack of biological markers [7]. ADHD is a neurodevelopmental disease that is often associated with ASD, and it is characterized by inattention, hyperreactivity and impulsivity, which interfere with daily activities [8].

The literature data indicate a connection between ASD and AD [9], and the etiological associations between these diseases have been extensively investigated in the last two decades [10]. In fact, the immune dysregulation and inflammation associated with genetic and environmental factors seem to characterize the pathophysiological mechanisms of both conditions [11].

Currently, the mechanisms that seem to be involved in the etiopathogenesis of both conditions include the following:

- Genetic predisposition: Signal transducer and activator of transcription 6 (STAT6) is a possible common signaling pathway-y that supports the hypothesis of an etiological correlation between atopy and neurodevelopmental disorders. Genetic variants of STAT6 are associated with atopy due to its major role in the regulation of the Th2 immune response. On the other hand, STAT6 is also expressed in the central nervous system and plays a key role in the pathogenesis of some neuropsychiatric conditions, including ADHD, which in turn is generally comorbid with ASD [9]. Other gene mutations involved in AD pathogenesis have also been observed in ASD, such as GATA3 and ADBRD2. In addition, it has been hypothesized that microRNA (miRNA) also plays a fundamental role [12].
- The production of pro-inflammatory cytokines (IL-6 and TNF alpha), which can cross the blood–brain barrier and activate neuroinflammatory mechanisms. The dysregulation of these cytokines is partially explained by alterations in the microbiota that may be found in both diseases.
- Mast cell activation, responsible for the release of mediators involved in the disruption of the blood–brain barrier and subsequent brain damage.
- The production of auto-antibodies against brain antigens (anti-MBP and anti-MAG) secondary to exposure to allergens. In fact, allergic responses are linked to an increased production of Th2 cells, which stimulate B lymphocytes to produce antibodies against allergens. In children with ASD, these could cross-react with sequence homologies with the brain, causing neuronal damage.
- Maternal and neonatal vitamin D deficiency [10].

However, despite the increasing importance of the link between skin diseases and mental disorders [13] and the increasing focus on understanding these pathologies, uncertainty lingers over multiple aspects. A better comprehension of the factors that characterize and influence the association between ASD and AD would be extremely useful for future studies, which could facilitate early diagnosis and prompt intervention in autistic patients, thus drastically improving their prognosis.

**Figure 1:**
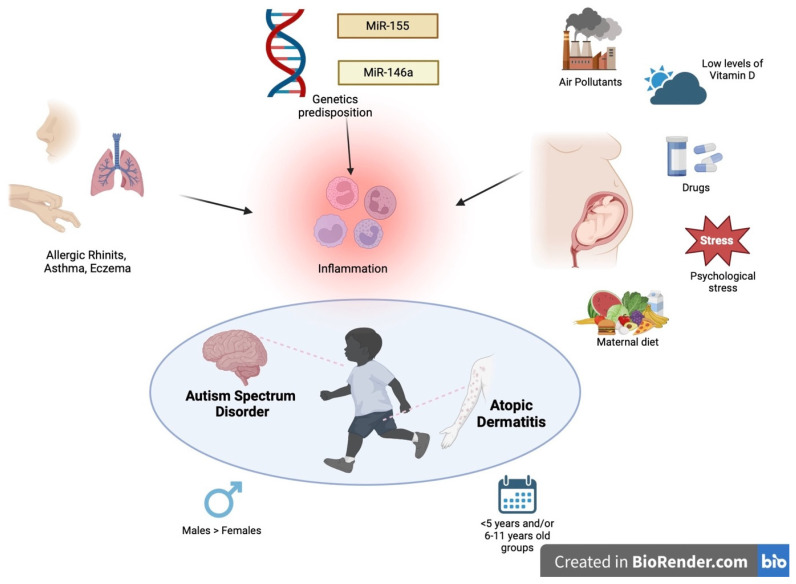
Common role of environmental (pregnancy, air pollutants, drugs, stress, diet), genetic predisposition and clinical co-factors (male sex and >5 years and/or 6–11 years old groups) in the production of inflammation which cause the onset and severity of the clinical course of patients affected by ASD and AD.

The aim of this review is to investigate and present the data available in the literature on the clinical features and alleged risk factors that could predict the onset of ASDs and/or AD or worsen their prognosis in the context of comorbidity. Our study focused on finding answers to seven questions, which have not been answered before and, above all, have not been brought together in the same work:

Does the exposome favor the onset of both autism and atopic dermatitis before, during and after pregnancy?
Does atopic eczema influence the severity of autism and vice versa?
Is there a correlation between the age of occurrence of both AD and ASD?
Can the presence of AD influence the onset of ASD/ADHD?
Are there gender differences between AD and ASD patients?
Are there comorbidities shared by both AD and ASD patients?
Are there genetic or epigenetic factors shared by both diseases? (Figure 1).

## 2. The Common Role of the Exposome in AD and ASD

Given that the brain and epidermis share the same embryologic origin, Shin et al. hypothesized that both tissues are susceptible to common insults; thus, neurotoxins that cause ASD should also attack the epidermis. In addition, pro-inflammatory cytokines derived from the stimulation of the neonatal skin by insults could traverse the infant’s immature blood–brain barrier, initiating or amplifying neuroinflammation [14]. Therefore, in these multifactorial diseases, exposure to environmental factors can have different effects depending on the genetic background. Some gene variants in ASD have been found to confer vulnerability to certain environmental stressors [15]. Environmental exposure may also play a role in AD through epigenetic alterations, including microRNA and DNA methylation, modulating transcript levels of genes involved in epidermal differentiation and innate immunity [16].

Repeated exposure to airborne pollutants (e.g., particulate matter, traffic-related air pollution, tobacco smoke and heavy metals) can result in an immune dysregulation that favors allergic reactions that may contribute to the pathogenesis and severity of atopic dermatitis [17,18]. At the same time, exposure to heavy metals during pregnancy may lead to neurodevelopment deficits. In fact, Skogheim et al. [19] and Pham et al. [20] reported a correlation between levels of metals during gestation and ASD in children.

Phthalates (i.e., DEHP [di-(2-ethylhexyl) phthalate]) are ubiquitous environmental contaminants used as plasticizers, solvents and additives in many consumer products [21]. They are suspected to be endocrine disruptors in susceptible individuals [22], and prenatal or perinatal phthalate exposure has been reported to decrease child mental development, interfering with cellular proliferation and lipid metabolism in the brain [23] through epigenetic modification [24]. Testa et al. evaluated the levels of DEHP metabolites in children with ASD, and demonstrated an association between phthalates and the disease [25]. These findings were also confirmed by Zhang et al. [26] and Nadeem et al. [27] in recent publications. Phthalates also have an adjuvant effect on allergen-related immunoglobulin production [28], enhancing allergic responses through the release of IL-4 from Th2 cells [29]. Several studies have revealed an association between phthalates and atopic dermatitis [30], showing that DEHP enhances atopic dermatitis-like skin lesions [28,29].

Perinatal exposure to environmental emissions of nitrous oxide (N_2_O) from agricultural and combustion pollutants have been found to be more frequent in subjects with ASD compared with control groups [31]. Fluegge suggested that a possible explanation for the association between AD and ASD could be the gestational exposure to N_2_O, which increases opioidergic activity and reduces vitamin D levels [32]. In fact, N_2_O could reduce the amount of solar radiation and thus reduce the absorption of vitamin D in the mother and thereby in the neonate [10]. Vitamin D plays an essential role in the skin barrier function and in the reduction of skin inflammation [33]. However it also plays a role in myelination and is considered a neuroactive steroid affecting brain development and function [34]. Meta-analyses have reported a lower serum 25(OH)D concentration in the overall adult and pediatric AD and ASD population than in healthy controls [34,35]. In fact, several studies have documented the aggravation of AD in winter and the improvement in both diseases after vitamin D supplementation, and decreased exposure to solar UVB might increase the risk of ASD [34,35,36,37].

Maternal folate status has also been linked to the risk of ASD and AD. Several studies suggest that mothers who use folic acid supplementation are less likely to have offspring with ASDs [38] or AD [39]; however, others have hypothesized an opposite relationship [40]. 

Maternal diets during pregnancy have been studied to determine possible associations with both diseases. because essential nutrients, which include carbohydrates, proteins, lipids, vitamins and minerals, are transferred from the mother to the fetus through the placenta during gestation and play an important role in fetal development [41]; however, no significant evidence has been found [41,42].

Valproic acid (VPA) is an anti-epileptic drug prescribed for women of child-bearing age, and its use is associated with an increased risk of congenital malformations and impaired cognition [43] and with severe hypersensitivity reactions [44,45,46]. Prenatal exposure to VPA is linked to a significant increase in the risk of ASD (approximately eight times higher than that of the general population) [47,48]. For the first time, Shin et al. assessed AD-like changes in the skin of neonatal offspring of valproic acid-exposed mid-trimester pregnant mice occurring by day one postpartum. The temporal co-emergence of AD and ASD was correlated with Th2 cytokine markers (i.e., IL-4, 5 and 13) and mast cells in the skin, followed only later by the appearance of neuroinflammation, supporting a possible skin → brain pathological sequence. The absence of injury in other tissues is linked to the susceptibility of these two embryologically-linked tissues to a common toxic insult, which explains the association of AD with a subset of ASD patients [14].

## 3. The Relationship between ASD and AD Severity

### 3.1. Studies Investigating Autism Severity in Patients with Eczema

A series of case–control studies by Mustafa et al. [49,50,51] highlighted an increased prevalence of different allergic manifestations (including atopic dermatitis) in patients with severe autism (defined by the Childhood Autism Rating Scale [52] ≥37). In addition, a 2013 population-based epidemiological study on children aged between 3 and 5 years by Shibata et al. [53] examined the association between autism severity (measured through the Japanese version of the Autism Screening Questionnaire, ASQ) and the presence of allergic symptoms. The overall ASQ score was not found to be significantly associated with the presence of symptoms; however the presence of eczema was associated with certain core autistic behavioral symptoms used as subscale items in the questionnaire. The most recent study to date on the subject was conducted in 2022 by Jameson et al. [54], who assessed the severity of autism with the Autism Diagnostic Observation Schedule, Second Edition (ADOS^®^-2) calibrated severity scores (CSSs) [55] in patients with and without atopic complaints. The results showed that children with atopy were 2.3 times more likely to present symptoms classified in the ADOS-2 highest severity level bracket and 2.9 times more likely to show social impairments. They also compared the atopic children in the eczema and asthma/allergies subgroups and observed a significant difference in a one-tailed test (*p* = 0.034 vs. two-tailed test: *p* = 0.069).

### 3.2. Studies Investigating Eczema Severity in Patients with Autism

In 2013, Yaghmaie et al. performed a cross-sectional survey study using data on children aged 0–18 from the 2007 US National Survey of Children’s Health. They found a significant dose-dependent relationship between the reported severity of eczema (assessed by the patient’s parent/guardian) and the reported prevalence of mental disorders, including autism [56]. Therefore, another study showed how alexithymia was more common among severe AD patients (43.6%) compared to mild AD patients (15.6%) and correlated with itch intensity and sleep disturbances [57]. In 2016, Liao et al. conducted a population-based longitudinal cohort study on children with an early AD diagnosis (under 2 years of age), whose data were obtained from Taiwan’s National Health Insurance Research Database (NHIRD). The authors assessed the severity of the eczema based on the number of clinical visits for AD the patients underwent in their first two years. The HR of ASD was found to increase with the increasing number of visits, and in particular, the risk was significantly higher for children who required further visits after 2 years of age [58]. On the other hand, in 2022, Wan et al. investigated the risk of major neuropsychiatric disorders in children with AD under the age of 18 from the UK Health Improvement Network (THIN) database. The study found no overall association between eczema and autism, thus highlighting an increased risk of autism (HR 1.25) only in patients with moderate eczema. In this case, the severity of eczema was estimated based on the treatment received by the patient (e.g., moderate eczema is defined by the use of potent topical corticosteroids or topical calcineurin inhibitors, whereas severe eczema is defined by the need for systemic immunosuppressants, phototherapy or dermatological referrals) [59] (Table 1).

To the best of our knowledge, studies analyzing the correlation between AD and ASD severity using validated physician-reported outcome measures for eczema such as EASI (Eczema Area and Severity Index) or SCORAD (SCORing Atopic Dermatitis) are currently lacking. In the studies by Mustafa et al. [49,50,51], eczema severity was assessed through the objective SCORAD. However, unfortunately, no separate analyses were performed for the different atopic diseases, thus mixing cutaneous and non-cutaneous infections. The authors found no significant correlation between autistic severity and allergy severity (*p* > 0.05).

Interestingly, the severity of both autism and eczema have been shown to correlate with the levels of some common pro-inflammatory cytokines such as TNF-alpha [60,61,62], which has been shown to play a role in the development of both diseases [63].

## 4. Age of Onset in Patients with Comorbid ASD and AD

The temporal relationship between the occurrence of ASD and eczema is poorly understood. To the best of our knowledge, only two studies are available that performed a stratified analysis by age. In the aforementioned 2016 study by Liao et al. [58] on Taiwanese children with AD onset under two years of age, the authors only performed a stratification comparing patients with an AD diagnosis under the age of one and between one and two years of age. The adjusted HR was slightly higher in patients with an earlier diagnosis, but not significantly. In the aforementioned 2022 population-based cohort study by Wan et al. on children in the UK aged 0–18, the authors also performed a substratification by age of the AD population into three cohorts (≤5, 6–11 and 12–17 years old). These authors found that patients in the ≤5 years old and 6–11 years old groups had a higher risk of developing autism (adjusted HR of 1.05 and 1.11, respectively) [59].

## 5. The Role of AD in the Onset of ADHD/ASD

The prevalence of ADHD and ASD has increased steadily over the past few decades [64,65]. The correlation between atopic dermatitis and ADHD/ASD has been studied for many years, but the data available in the literature are still contrasting [10,66,67]. The causes for the conflicting reports are probably related to differences in the study population, follow-up period, diagnostic criteria and the different allergic disorders that were examined [68].

Buske-Kirschbaum et al. proposed different mechanisms to explain the association between AD and ADHD: (1) atopy causes an exaggerated release of inflammatory cytokines which interfere with the maturation of the prefrontal cortex regions and neurotransmission involved in ADHD; (2) eczema is typically associated with high levels of psychological stress; (3) shared genetic factors; and (4) prenatal stress [69]. Elevated cytokine levels have also been reported in children with ASD, and a correlation between proinflammatory cytokines and symptom severity has been observed in ADHD [70,71]. Two systemic reviews have shown a correlation between AD and ADHD and between AD and ASD [72,73]. Evidence of an association between allergic diseases and ADHD/ASD in children has also been reported in several longitudinal studies.

For example, in a cohort of 770 children in Germany, Genuneit et al. [74] reported a significant association between early AD in the first 4 years of life and the development of ADHD by the age of 8 years. In another longitudinal study conducted in Taiwan, including 18,473 children aged between one month to three years with AD, the authors found a significant relationship between AD and ADHD compared to healthy controls without AD. The presence of asthma and allergic conjunctivitis further increased the risk of developing ADHD compared to those with AD alone [75]. Another study conducted in Taiwan also reported a correlation between being diagnosed with AD under the age of two and an increased risk of ADHD and ASD. This risk increased further in children with severe AD and concomitant atopic respiratory disease [58].

## 6. Gender Differences between Atopic Dermatitis and ASD

A 2008 study conducted in the Netherlands revealed a slightly higher prevalence of AD in boys compared to girls (8.7% vs. 5.6%) among children under four years of age [76]. On the other hand, studies on an adult population showed a higher prevalence of AD in females: 5.7% and 8.1% for men and women in Japan, and 6.04% [77] and 8.01% for men and women in Europe and the USA, respectively [19]. A 2022 Swedish population cohort study by Johansson et al. also evaluated differences in the location of AD lesions between sexes. The extremities, hands, trunk and scalp were the most commonly affected sites for both men and women. A difference was also found in the reporting of lesions on the groin and pubic regions, which were affected in 14.1% of males compared to 7.9% of females [20].

After puberty, the secretion of sex hormones from ovary, testis and adrenal glands increases substantially. The onset of atopic dermatitis in adult females seems to be linked to the influence of sex hormones on the immune system and skin permeability. In fact, estrogen and progesterone enhance the activity of Th2 and regulatory T cells, while androgens suppress Th1, Th2 and Th17 activities, and the skin barrier is enhanced by estrogen and compromised by androgens and progesterone [78]. In addition, females have a higher number of CD4+ CD19+ lymphocytes and therefore a higher number of IgE, IgG, IgM and IgA antibodies. In contrast, umbilical cord blood IgE levels in male infants are higher than in female infants [79].

The literature data show that ASD is 2–3 times more predominant in males compared to females [80]. This is partially explained by the fact that ASD seems to be under-recognized in girls and women, with a higher average age at diagnosis compared to males [81,82,83]. However, being female has also been shown to protect against autistic impairment [84]. The higher preponderance of ASD in males could be due to genetic factors, such as sex-specific single nucleotide polymorphisms and variants, or the alleged protective role of a second X chromosome against neurological spectrum disorders [85].

As with AD, the gender difference in autism may be linked to the immature brain’s exposure to male hormones. A report by Baron-Cohen et al. directly compared the fetal sex steroid levels of boys who later received a diagnosis of ASD to those of typically developing controls. Individuals with ASD had higher amniotic levels of progesterone, 17α-hydroxy-progesterone, androstenedione, testosterone, and cortisol in the first few weeks after birth, and these hormone levels remained elevated throughout childhood [86].

It is assumed that there are two converging factors in relation to the amygdala that contribute to the risk of ASD in genetically predisposed individuals. These are boys’ exposure to high testosterone levels, and antenatal, perinatal and early childhood stress mediated by an increase in androgens [87]. Evidence of the effects of testosterone also comes from studies on girls with congenital adrenal hyperplasia or polycystic ovary syndrome. The correlation in these populations of increased testosterone levels with autistic trait scores has been demonstrated in many studies [88]. Estrogen and oxytocin are protective for females. In fact, females during birth are exposed to higher levels of estrogen and lower levels of androgen. Oxytocin has been shown to contribute to pro-social behaviors [89].

## 7. Common Comorbidities between AD and ASD

A study conducted on 50 children with ASD showed an association between autism and allergies (respiratory allergy and atopic dermatitis), as allergies were found in 57.7% of children with autism (80% if considering only females) and in 80% of patients with severe autism. Autistic children with an allergy had a higher prevalence of neurological gastrointestinal symptoms, neurological manifestations, autistic regression, mental retardation, sleep abnormalities and electroencephalography abnormalities [50]. Mostafa et al. [49] also investigated the link between allergic manifestations and elevated serum levels of brain-specific autoantibodies in autistic children as a marker of potential CNS autoimmunity. Mostafa et al. reported an association between the presence of one or more allergic manifestations and the severity of autism. In fact, the frequency of allergic manifestations in patients with severe autism (61.5%) was significantly higher than in children with mild to moderate autism (25%) [49].

In a cohort of 14,812 atopic subjects, Chen et al. [90] demonstrated that atopic status in early childhood increased the risk of ASD. Among all the atopic individuals, 12.2% were diagnosed as having one atopic comorbidity, 37.2% had two atopic diseases, 33.4% had three atopic diseases, and 17.1% had four atopic comorbidities. Data from these subjects revealed a correlation between the number of atopic comorbidities and an increased risk of ASD.

Using data from the Taiwan National Health Insurance Research Database (TNHIRD), Lin et al. described a higher probability of presenting with atopic diseases (asthma, allergic rhinitis, allergic conjunctivitis, atopic dermatitis) among children with the association of ASD and ADHD (458 children, OR: 2.26) compared to that presented by children with only ADHD (5386 children, OR 1.81) or only ASD (578 children, OR: 1.24) [91]. From the same source, Lee et al. showed an increased risk of developing ADHD (HR 2.92) or ASD (HR: 8.9). The risk of developing ADHD and ASD was also higher in AD children presenting three comorbidities: allergic rhinitis, allergic conjunctivitis and asthma [74].

**Figure 2:**
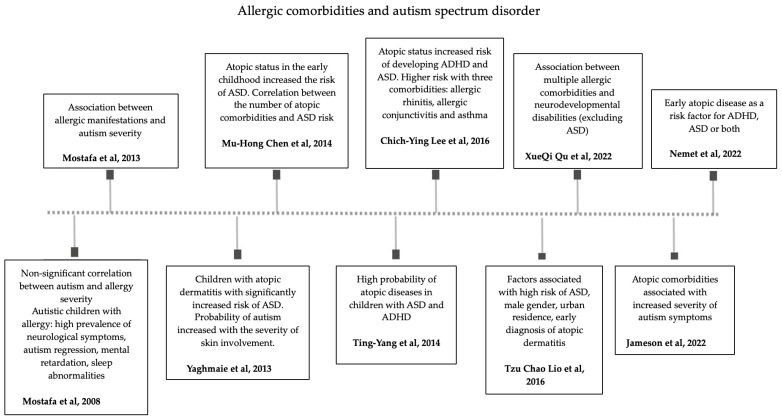
The timeline of research on the link between atopic comorbidities and autism spectrum disorders [49,50,51,54,56,58,75,90,92,93].

Interestingly, Xueqi Qu et al. showed an association between multiple allergic comorbidities and neurodevelopmental disabilities, excluding ASD [92]. In a large retrospective study conducted on 117,022 consecutive children registered in the pediatric database of Clalit Health Services (2000–2018), the early development of atopic diseases and more than one comorbidity were recently described as risk factors for presenting ADHD, ASD or both [93] (Figure 2).

## 8. Link between the Genetics and Epigenetics of Autism and Atopic Dermatitis

Autism is considered as the most heritable neurodevelopmental disorder, based on a 70–90% concordance rate in identical twins, which highlights the importance of genetics [94]. Although numerous studies have demonstrated the heritability of ASD, not much is known about specific genetic variants (single-nucleotide or copy number variants) [95]. Some ASD-associated CNVs are inherited from an unaffected parent; however, in 2007, Sebat [96] and subsequent studies demonstrated an association between de novo copy number variants and ASD. The presence of these copy number variants may be considered as risk factors for the development of sporadic ASD [97,98]. The first demonstration of the genome-wide significant association of common variants with ASD was reported by Wang et al. [99] in an early genome-wide association study (GWAS) of 4300 children affected with ASD and 6500 control subjects. A strong association was found with six single-nucleotide polymorphisms (SNPs) located between the cadherin 10 (CDH10) and cadherin 9 (CDH9) genes located on chromosome 5. These genes encode neuronal cell adhesion molecules. Butler et al. [100] collected a list of 800 genes, including members of the neuroligin, neurexin, GABA receptor, cadherin, and SHANK gene families, that were implicated in ASD. A gene–environment interaction has been shown to result in epigenetic abnormalities. These epigenetic alterations change gene expression levels without changing genome sequences. Epigenetic mechanisms have been found involving DNA methylation, transcriptional regulation and post-translational changes in histone proteins [101]. Epigenetic mechanisms for AD development, including genomic DNA modification and microRNA post-transcriptional regulation, have also been explored. Candidate gene association studies indicate that mutations in the filaggrin (FLG) gene are the most significant known risk factors for atopic dermatitis (AD), and genes in the type 2 T helper lymphocyte (Th2) signaling pathways are the second most replicated genetic risk factors for AD. Additionally, gene profiling assays have shown that AD is associated with the decreased gene expression of epidermal differentiation complex genes and elevated Th2 and Th17 genes. The hypomethylation of TSLP and FCER1G in AD has been reported [102].

Studies have explored the role of miRNAs in several molecular and cellular mechanisms, including neurodevelopment, brain plasticity and immunity, in the etiopathogenesis of ASD [103]. It has been suggested that miRNAs are involved in the pathological process of atopic dermatitis [104]. According to the studies on miRNAs in AD and ASD, miR-146a has emerged as the most representative, and is upregulated in various neurodevelopmental disorders. miR-146a has been reported to be highly expressed throughout the cortex, hippocampus and amygdala, and to play a role in higher cognitive functioning [105]. In addition, it has been demonstrated that miR-146a overexpression in mouse primary cell cultures leads to impaired cognitive function, thus confirming the defective neural connectivity typical of ASD, in turn modifying synaptic transmission at the CNS level [105].

The function of miR-146a in the regulation of inflammatory processes could partially explain the hypothetical association between the common pathophysiological pathways of these two conditions. It is in fact evident that increased miR-146a expression is present in the lesioned skin of AD patients, as it inhibits nuclear factor κ B (NF-κB)-mediated proinflammatory cytokines and chemokines [106].

The role of miR-155 in ASD is not yet known, whereas in AD, MiR-155 is implicated in the differentiation of T helper type 17 (Th17) cells [107].However, both miR-146a and 155 appear to be involved in this common pathogenetic pathway, and the current data support future applications for miR-146a, both as a biomarker and as a target for therapy [12]. Beyond miRNAs in the pathogenetic pathway, some evidence suggests that proinflammatory cytokines play an important role in the development of ASD during the atopic response. It is believed that dysregulation of the inflammatory pathway may impact the central nervous system and be involved in the mechanisms of this neurodevelopmental condition. The atopic response in AD begins with the uptake and processing of allergens by Langerhans cells (LCs) after penetrating the compromised epithelial barrier. The activated LCs then migrate to the skin, and IL-4 promotes Th2 differentiation. Keratinocytes produce chemokines, which recruit Th2 cells and eosinophils to the skin. This enhanced inflammatory response may complicate AD with bacterial infections [10].

Staphylococcal exotoxins activate LCs to produce inflammatory mediators, including IL-1b and tumor necrosis factor TNF-a after penetration through the compromised epithelial barrier. In fact, it seems that proinflammatory cytokines may penetrate the blood–brain barrier and activate neuroimmunological mechanisms involving specific neural circuits (i.e., the anterior cingulate gyrus and insula) related to behavioral and emotional modulation. Wei et al. suggested that IL-6 was significantly increased in the cerebellum of ASD subjects and altered neural cell adhesion, migration and synaptic formation [108].

Recently, a meta-analysis identified several cytokines, including IL-6, IL-1β, IL-12p70, macrophage migration inhibitory factor (MIF), eotaxin-1, monocyte chemotactic protein-1 (MCP-1), IL-8, IL-7, IL-2, IL-12, tumor necrosis factor-α (TNF-α), IL-17 and IL-4, as potential biomarkers for ASD. These cytokines are also associated with AD, suggesting that variations in their levels in early life may contribute to the development of ASD. The role of Th2 cytokines as a potential link between the two conditions is currently being investigated [11].

## 9. Conclusions and Future Perspectives

The early diagnosis of ASD is of the utmost importance, to improve the long-term outcomes of the patient (adaptive and social behavior, language, cognition) and the quality of life of their caregivers through early interventions. However, the average age for the diagnosis of ASD is still between 4 and 5 years, despite the fact that a reliable diagnosis can be achieved by age two and that standardized screening methods are available for younger children [109]. The identification of risk factors and clinical features associated with ASD could help not only in the creation of diagnostic/screening tools for use in clinical practice, but also in gathering data for machine learning models. These models could provide a substantial aid in the investigation of neurodevelopmental disorders in the early stages and predicting clinical outcomes such as the ADOS-2 score [110]. The research gaps highlighted in this paper, such as the uncertain role of some environmental risk factors or the lack of clinical studies using standardized severity scores for both eczema and ASD, should incentivize future research aimed at addressing these shortcomings and providing data for more reliable statistical algorithms.
**Key Points**Atopic dermatitis is the most common chronic pediatric skin disorder, characterized by itchy eczema.Autism spectrum disorders refer to a group of neurodevelopmental diseases that affect children’s interaction and communication skills.The brain and epidermis, which share the same embryological origin, may be susceptible to common environmental insults, such as pollutants, N_2_O, drugs, and maternal diet.The severity of both autism and eczema is correlated with the levels of common pro-inflammatory cytokines (TNF-alpha).Children with atopic dermatitis who are ≤5 years old and between 6 and 11 years old have a higher risk of developing autism.The presence of allergic comorbidities increases the risk of developing AD.Males have a greater risk of developing both autism and atopic dermatitis.The identification of risk factors and clinical features associated with both ASD and AD can help in the creation of diagnostic/screening tools for use in clinical practice and could help in predicting clinical outcomes.

## Figures and Tables

**Table 1:** Comparison of the different methods used in clinical studies to assess the severity of autistic spectrum disorders and atopic dermatitis in comorbid patients.

Clinical Studies on ASD Severity in AD Patients	Method Used to Assess ASD Severity
Mostafa et al. (2008–2013) [49,50,51]	Japanese version of the Autism Screening Questionnaire (ASQ)
Shibata et al. (2013) [53]	Childhood Autism Rating Scale (CARS)
Jameson et al. (2022) [54]	ADOS-2 calibrated severity scores
Clinical studies on AD severity in ASD patients	Method used to assess AD severity
Yaghmaie et al. (2013) [56]	Assessed by the patient’s parent/guardian by posing the question: ‘‘Would you describe [his/her] eczema or skin allergy as mild, moderate, or severe?’’
Liao et al. (2016) [58]	Number of clinical visits for AD the patients underwent under the age of two (1, 2–3, or 4 or more)
Wan et al. (2023) [59]	Based on treatment used, AD was classified as - Moderate: use of a second potent topical corticosteroid within 1 year, or a first topical calcineurin inhibitor - Severe: usage of phototherapy or systemic immunosuppression or need for dermatological referral - Mild: patients who were not classified as either moderate or severe

Abbreviations: ASD: autistic spectrum disorder; AD: atopic dermatitis; ADOS-2: Autism Diagnostic Observation Schedule, Second Edition.
